# The Washout of Hepatocellular Carcinoma at Portal Venous Phase vs. Equilibrium Phase: Radiological and Clinicopathological Implication

**DOI:** 10.3390/cancers17193195

**Published:** 2025-09-30

**Authors:** Kengo Yoshimitsu, Akihiro Nishie, Yukihisa Takayama, Shinji Tanaka, Keisuke Sato, Kousei Ishigami

**Affiliations:** 1 Department of Radiology, Faculty of Medicine, Fukuoka University, Fukuoka 814-0180, Japan; ytakayama@fukuoka-u.ac.jp (Y.T.); 308104@gmail.com (S.T.); stksk310@gmail.com (K.S.); 2 Department of Radiology, Graduate School of Medical Science, University of the Ryukyus, Okinawa 903-0215, Japan; nishie_a@cs.u-ryukyu.ac.jp; 3 Department of Clinical Radiology, Graduate School of Medical Sciences, Kyushu University, Fukuoka 812-8582, Japan; ishigami.kosei.581@m.kyushu-u.ac.jp

**Keywords:** hepatocellular carcinoma, washout, portal venous phase, equilibrium phase, clinico-pathological implication

## Abstract

**Simple Summary:**

Portal venous and equilibrium (late) phase washouts are recognized features of hepatocellular carcinoma (HCC), yet they indicate different intratumoral conditions. Positive portal venous washout has been associated with high-grade tumors, poor postoperative survival, and PD-L1 or VETC positivity. Conversely, lack of washout at equilibrium has been linked indirectly to biliary/stem cell subtypes, which are biologically aggressive and often show an immune hot microenvironment. Although these two patterns arise from distinct pathophysiologies, both are closely related to tumor aggressiveness and the microenvironment, factors influencing prognosis, and response to systemic therapy. When evaluated together and integrated with other clinico-radiological features, more accurate prediction of survival or treatment outcome may become available.

**Abstract:**

Portal venous or late (equilibrium) phase washout is one of the well-known major imaging features of hepatocellular carcinoma (HCC). However, these two washouts stand for distinct intratumoral pathophysiological states and should be considered separately. Positive portal venous phase (PVP) washout has been shown to be related to high grade HCC, poor post operative survival rate, and positive PD-L1 or VETC. In contrast, there is indirect evidence that negative washout at equilibrium or late phase (EqP) may be related to biliary/stem cell subtype, which is biologically aggressive, and associated with an immune hot tumor microenvironment. Thus, although these two washouts represent different intratumoral pathophysiological conditions, both are closely related to biological aggressiveness or tumor microenvironment, which may be associated with the response to systemic therapies or post-surgical survival. In contemporary practice, gadoxetate-enhanced MRI restricts washout assessment in the PVP, whereas extracellular agent CT permits assessment in both the PVP and EqP; accordingly, this review addresses PVP washout on CT or extracellular agent MRI, PVP washout on gadoxetate-enhanced MRI, and EqP washout on CT. When washout information is integrated with other clinico-radiological features, more precise prediction of patient survival or response to systemic therapies would become possible in the future.

## 1. Introduction

Arterial phase hyperenhancement (APHE) and washout at either the portal venous phase (PVP) or late/equilibrium phase (EqP) are the two major imaging features of hepatocellular carcinoma (HCC) [1].

APHE is generally considered to represent an arterial-dominant blood supply for HCC, which develops over the course of multistep-hepatocarcinogenesis [2,3,4,5,6,7,8,9,10,11,12,13,14,15,16,17,18].

In contrast, the biophysical and physiologic mechanisms that underlie the phenomenon of “washout” have been less thoroughly investigated in detail so far [19,20,21,22,23,24]. Simply put, “washout” may be defined as “relative hypoattenuation (on CT) or hypointensity (on MRI) of an HCC lesion as compared to the surrounding background liver (BGL)”. However, the PVP and EqP represent markedly different hemodynamic conditions: the PVP is a period during which the hemodynamics within the liver is dramatically changing, whereas the EqP is a static period at which the hemodynamics is stable. For this reason, “washout” occurring at PVP vs. that at EqP should be understood as representing distinct imaging and possibly pathophysiologic phenomena and should be interpreted separately.

In current clinical practice, the majority of liver MR imaging employs gadoxetate (also known as EOB, e.g., Gd-EOB-DTPA) enhancement (henceforth EOB-MRI), in which “washout” assessment is confined only to the PVP [1] because there is no EqP for EOB-MRI in the strictest sense, whereas CT with extracellular contrast medium containing iodine allows for assessment both on the PVP and the EqP.

The purpose of this review article is to comprehensively describe the current understanding status and clinico-radiological implication of “washout” of HCC by dividing it into three categories, namely that on the PVP of CT or extracellular contrast medium enhanced MRI (ECCM-MRI) (Section 2.1), that on the PVP of EOB-MRI (Section 2.2), and that on the EqP of CT (Section 3). And finally, the summary, current limitations, and future study directions will be provided in Section 4.

## 2. Washout at the Portal Venous Phase

### 2.1. Washout at the PVP of CT or ECCM-MRI

As noted above, washout of HCC either at the PVP or at the EqP is considered a major feature in Li-RADS; however, washout at PVP is not actually observed in all cases. According to our previously published data, out of HCC showing APHE, only about 20% showed washout at the PVP of CT [20], whereas at EqP, more than 90% displayed washout. Similar results have been reported so far by several researchers [21,22,23].

**Figure 1:**
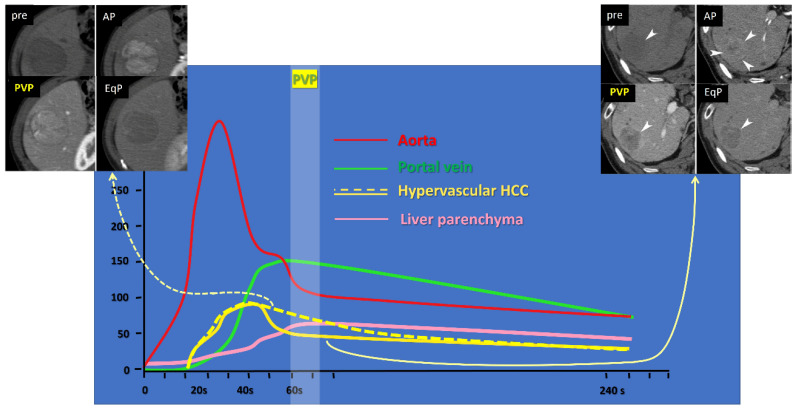
Time-density curve of upper abdominal dynamic CT after intravenous iodine contrast medium injection. Yellow lines indicate curves for hypervascular hepatocellular carcinomas (HCCs), showing higher density than the background liver parenchyma (pink line) at the arterial phase (AP, around 30–40 s); the solid yellow line represents HCC with fast intratumoral flow, showing a rapid attenuation drop at the portal venous phase (PVP, around 60–70 s), lower than the liver parenchyma (pink line); the dotted yellow line represents HCC with slow intratumoral flow, showing persistent enhancement, higher than the liver parenchyma (pink line). Representative cases are shown at the right upper corner, and the left upper corner, corresponding to the solid and dotted yellow lines, respectively. EqP stands for equilibrium phase, set at 240 s in our institution.

Based on the hemodynamic change in the liver during intravenous bolus injection of ECCM, including iodine contrast medium for CT or gadolinium (Gd)-based ECCM for MR, it has been shown that the liver parenchymal enhancement in the PVP reaches its peak and then stabilizes approximately one minute after the commencement of ECCM intravenous injection (Figure 1). We therefore hypothesized that the different washout status of HCC at the PVP arises from factors on the HCC side. We thus correlated several pathological features of HCC to washout status at the PVP in the previous study [19,20], and found that positive washout of HCC at PVP is closely related to higher histological grade or poorly differentiated HCC (pHCC). Several other investigators have reported similar results [21,22,23], namely, 60% to 70% sensitivity and accuracy, 80% to 90% specificity and positive predictive value, and 30–40% negative predictive value, when positive PVP washout of hypervascular HCC was considered as a sign of moderately to poorly differentiated HCC, except for one CT paper [20]. In this particular paper [20], sensitivity, accuracy, and negative predictive value are very low, down to around 20%, whereas specificity and positive predictive value were 100%. This disparity likely results from the very early timing of the PVP in that study, namely, its PVP was obtained only 20 s after the arterial phase (AP), whereas other studies [21,22,23] obtained PVP images around 30 s after AP. Too early PVP acquisition may result in incomplete washout, compromising sensitivity, accuracy, and negative predictive value, while by contrast, enhancing specificity and positive predictive value. Thus, the PVP washout status of HCC appears to be influenced not only by the histological grades of HCC, but also at least partially by scanning protocol parameters such as timing.

**Figure 2:**
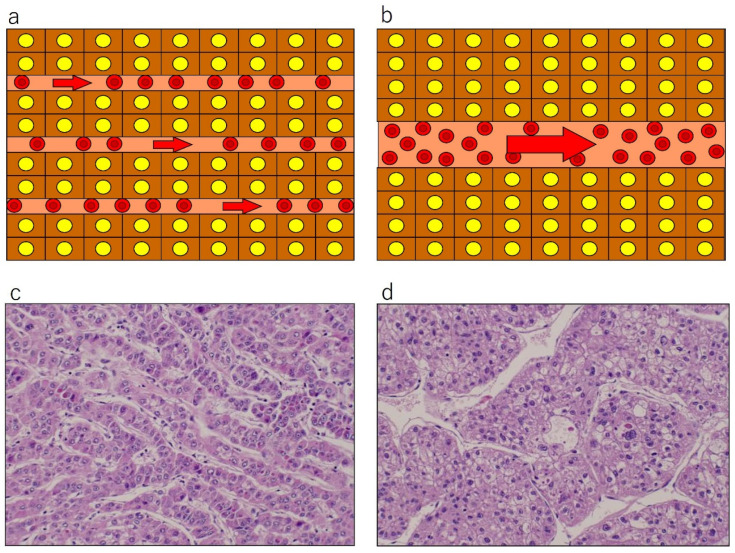
Schematic presentation (**a**,**b**) and actual pathological appearance (**c**,**d**): hematoxylin and eosin staining, original magnification ×400) of tumor plate architecture and sinusoid caliber in hepatocellular carcinoma (HCC). Well differentiated HCC (**a**,**c**) shows thin tumor plates and narrow and numerous tumor sinusoids, where resistance would be high and flow speed would be slow, under the Hagen–Poiseuille assumption. Poorly differentiated HCC (**b**,**d**) exhibits thick tumor plates and wider, fewer, and irregular-shaped tumor sinusoids, where resistance would be lower and flow would be fast. Reproduced from references [12,19].

**Figure 3:**
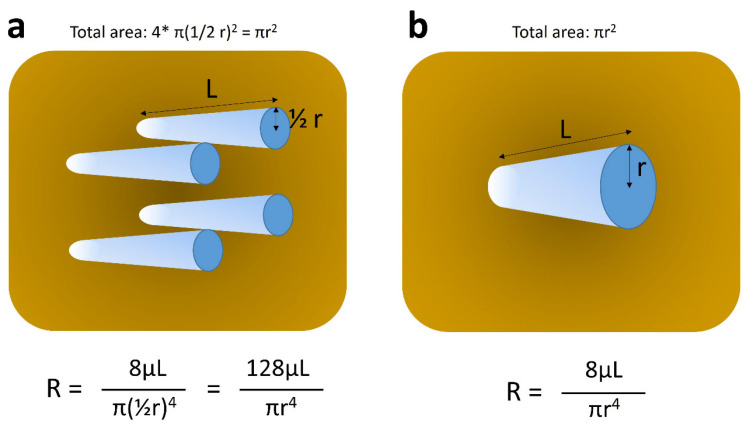
Schematic explanation of Hagen–Poiseuille’s law. Each tube represents pseudo-tumor sinusoid. (**a**) and (**b**) stand for relatively well and poorly differentiated hepatocellular carcinomas, which contain tumor sinusoids of small caliber and large number, and those of large caliber and small number, respectively. The sum of cross-sectional areas of tumor sinusoids is assumed to be identical between (**a**) and (**b**) (πr^2^), as per our previous observation [12]. Under these circumstances, the resistance of intratumoral flow is much higher (approximately 15 times) in (**a**), as compared to that in (**b**), leading to slower flow in (**a**) and faster flow in (**b**). R, resistance; r, radius of each tumor sinusoid; π, pi; μ, viscosity of the fluid; L, length of tumor sinusoid.

Taken as a whole, accumulated data suggest that positive PVP is more likely indicative of moderately to poorly differentiated HCCs, rather than well differentiated tumors. However, the precise mechanism behind this phenomenon remains incompletely understood. We speculated as follows [19,20]: most HCCs consist of tumor plates or trabeculae, mimicking normal liver structure, but they are much thicker in width, or more irregular in shape than their normal counterpart, and these tendencies increase as the histological grades of HCCs become higher. Our observation has shown that the total area of tumor sinusoids (vascular spaces between tumor plates, filled with tumor blood) is almost consistent regardless of the histological grades of HCCs [19,20]. This indicates that low grade or well differentiated HCCs (wHCCs) tend to have many thin tumor plates and many narrow tumor sinusoids, whereas high grade or pHCCs have thicker, more irregular tumor plates, and fewer but wider or larger tumor sinusoids (Figure 2). Under a model approximating intratumoral perfusion in accordance with Hagen–Poiseuille’s law, the flow resistance would be higher in lesions with many small-caliber sinusoids, and lower in those with fewer large-caliber sinusoids (Figure 3), leading to slower flow (and hence absent or less pronounced washout) within wHCCs and faster flow and rapid washout within pHCCs at PVP.

### 2.2. Washout at the PVP of Gadoxetate-Enhanced MRI (EOB-MRI)

Gadoxetate (Primovist^®^, Bayer HealthCare) is a hepatocyte-specific contrast agent and begins to be taken up by functioning hepatocytes approximately one minute after intravenous injection, i.e., roughly corresponding to the PVP [25]. In LIRADS v2, washout of HCC is restricted exclusively to the PVP, not the transient/transitional phase or the hepatobiliary phase [1]. Strictly speaking; therefore, washout of HCC as observed at the PVP of EOB-MRI is not identical to that seen on CT or ECCM-MRI. In other words, theoretically, there should be concerns that the PVP of EOB-MRI may be overly sensitive, or conversely less specific, in terms of the detection of pHCC, due to the active uptake of the contrast medium by the BGL. Nevertheless, several researchers have reported that positive washout at the PVP of EOB-MRI is closely related to the biological aggressiveness or higher histological grade of HCC [26,27,28], which are very similar to those seen for ECCM PVP washout.

Fujita et al. analyzed 345 surgically proven HCC patients and determined factors related to microvascular invasion (MVI) of HCC, which is a well-known prognostic factor after surgical resection or transplantation. They found that washout at the PVP of EOB-MRI is significantly related to poor differentiation of HCC and positive MVI, and multivariable analysis revealed that tumor size, positive PVP washout, and peritumoral hypointensity at HBP are independently significant factors related to MVI [26]. Tomino T et al. [27] analyzed 206 surgically resected HCC patients, in an attempt to clarify the prognostic impact of signal intensity of HCC at the PVP of EOB-MRI. They introduced SIRPP (signal intensity ratio at portal phase) as the ratio of signal intensity of HCC to that of the BGL, which is a quantitative index of “washout”. They found that low SIRPP value, roughly indicating positive washout, and high alpha-fetoprotein level are independently significant factors related to pHCC by multivariable analysis, and there were significant differences in overall survival and also in recurrence-free survival between high and low SIRPP groups (roughly corresponding to negative and positive washout groups). Furthermore, low SIRPP was an independently significant factor for overall survival after curative surgical resection of HCC [27]. The same group investigated the correlations among intraoperative indocyanin green fluorescence patterns, preoperative EOB-MRI features, and histological characteristics in 80 HCCs [28]. In their subanalysis, they found that low SIRPP, namely, the rough index of positive washout at the PVP of EOB-MRI, is significantly related to intratumoral CD8-positive T-cell (program cell death ligand 1/PD-L1) expression percentage, and also to vessels encapsulating the tumor cluster (VETC) percentage, in addition to poor differentiation of HCC and presence of MVI [28]. Because positive PD-L1 and VETC have both been known to be linked with responses to recent combination immunotherapy or to anti-vascular endothelial growth factor (VEGF) therapy, positive washout at the PVP of EOB-MRI could potentially serve as a predictive biomarker for response to these systemic therapies.

Thus, washout on ECCM PVP and EOB-MRI PVP can be regarded as broadly similar in their associations with tumor aggressiveness or as a potential predictor for response to systemic therapy.

**Figure 4:**
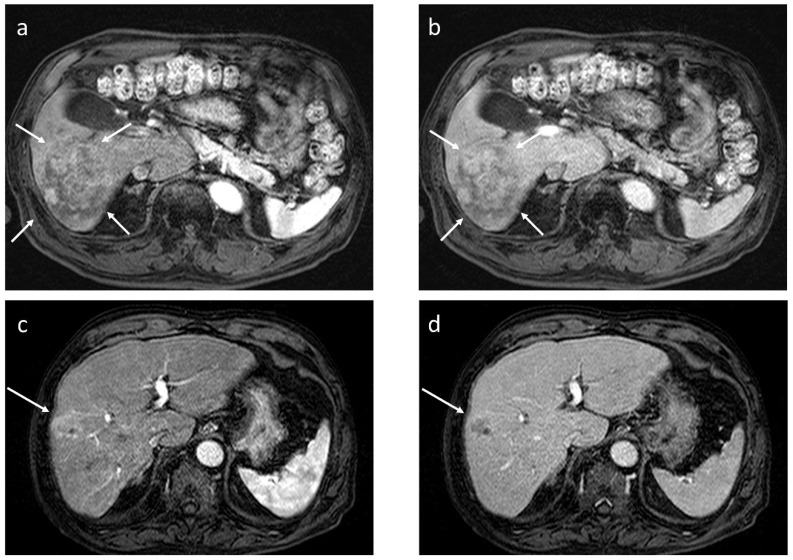
Dynamic gadoxetate-enhanced MRI in two representative cases ((**a**,**b**) and (**c**,**d**)). Both lesions exhibit arterial phase hyperenhancement (**a**,**c**), but in the former, there is no portal phase washout ((**b**) arrows), whereas in the latter, there is apparent portal phase washout ((**d**) arrow); the former pathologically turned out to be well to moderately differentiated hepatocellular carcinoma (HCC), and the latter to be poorly differentiated HCC.

As for the scanning protocol parameters, in all papers cited here [19,26,27,28], PVP was obtained 30 s after AP delay time; therefore, there was considered to be little concern about the bias originating from technical aspects when interpreting their data. Representative cases are illustrated in Figure 4.

### 2.3. Washout at the Equilibrium Phase of CT

**Figure 5:**
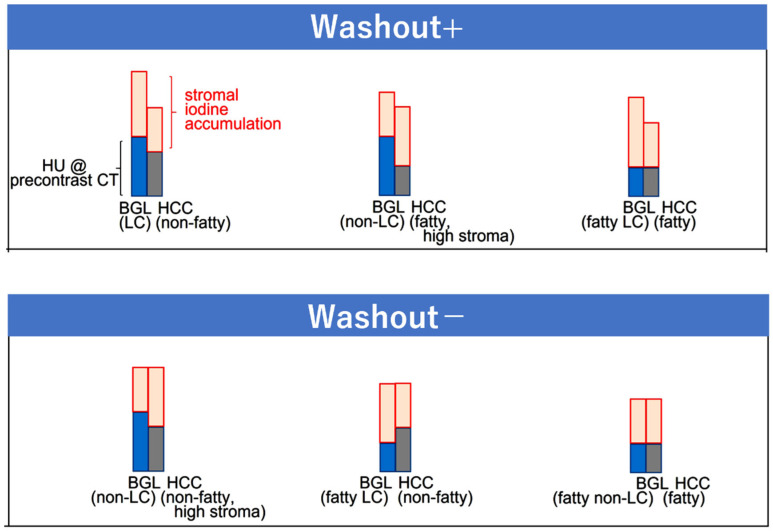
Scheme of washout patterns at the equilibrium phase (EqP) of CT. Washout status is determined by the relative difference between the density of hepatocellular carcinoma and the background liver, which is defined as the sum of plain CT density (blue and grey for BGL and HCC, respectively) and the degree of iodine accumulation (pink) at the EqP. The diagram shows representative combinations that yield positive or absent washout. HU, Hounsfield unit; BGL, background liver; HCC, hepatocellular carcinoma; LC, liver cirrhosis. Reproduced from reference [24].

Washout of HCC at the EqP is determined by the balance between the sum of precontract CT density and degree of iodine accumulation of HCC and that of the BGL at the EqP (Figure 5) [24]. In our previous study, multivariable analysis revealed that factors related to washout status at the EqP were extracellular volume fraction (ECV) and precontrast density (in Hounsfield units, HU) of both HCC and the BGL, which is quite reasonable because ECV is a standardized index reflecting the amount of iodine accumulation in extracellular compartments [17]. Specifically, ECV is defined as a sum of sinusoidal or intravascular space (IVS) and extravascular extracellular space (EES), in both of which extracellular contrast medium, such as iodine for CT, can exist at the EqP, and can be expressed as the following formula:

ECV_tissue_ = (1 − hematocrit) × ΔHU_tissue_/ΔHU_aorta_ (1)

where ΔHU represents the density difference between the EqP and precontrast phase (HU_Eq_−HU_precon_).

**Figure 6:**
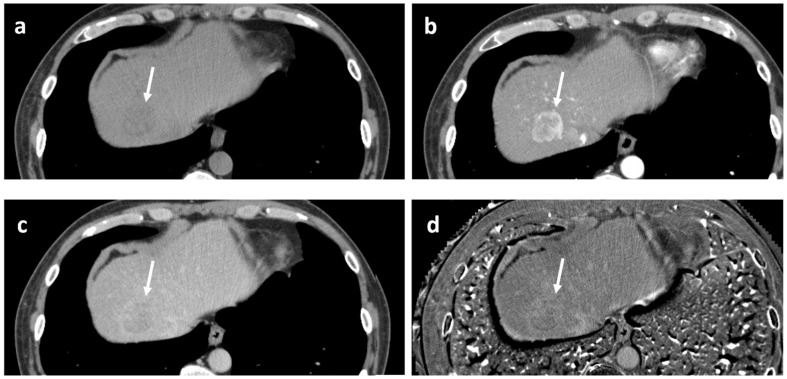
Example of positive washout case at the equilibrium phase. 53-year-old man with alcoholic liver disease. Pathologically, moderately differentiated hepatocellular carcinoma of confluent multinodular type, without complete fibrous capsule, was confirmed. Fatty change and lymphocytic infiltration were observed within the tumor. (**a**) Precontrast CT. The tumor exhibits low density (arrow). Measured density were 46 and 56 Hounsfield units for the tumor and the background liver, respectively. (**b**) Arterial phase CT. The tumor shows periphery-dominant enhancement (arrow). (**c**) Equilibrium phase CT. The tumor exhibits apparent “washout” (arrow). (**d**) Extracellular volume fraction (ECV) map reveals slightly higher value of the tumor, as compared to the background liver. Measured ECVs were 38.5% and 33.1% for the tumor and the background liver, respectively. In this case, although the ECV of the tumor is slightly higher than that of the BGL, it was considered that the lower precontrast density of the tumor, possibly due to fatty change, contributed to the positive EqP washout.

A representative washout positive case is shown in Figure 6.

It has been well established that ECV_BGL_ is a reliable marker of histological grades of liver fibrosis, since fibrosis primarily resides in EES of the liver parenchyma [29,30,31,32,33,34,35,36,37,38,39,40,41,42,43,44,45,46,47]. Although MR elastography (MRE) may offer superior accuracy for grading liver fibrosis on a patient-by-patient basis [40], ECV has advantages in the assessment of small focal areas such as HCC [24,46,47]. In HCC, pathological studies have shown that the majority of the constituents of tumor stroma are collagen or elastin [48], which are considered to exist also in the EES of HCC. Thus, ECV_HCC_ serves as a surrogate marker of the amount of intratumoral stromal component (extracellular matrix), which has been shown to be related to tumor microenvironment or stemness of the tumor [49,50].

**Figure 7:**
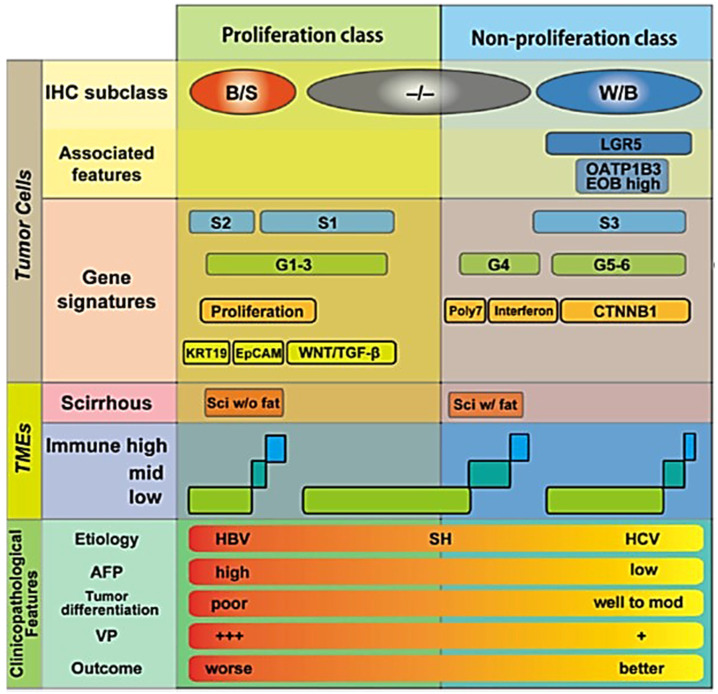
Details of the relationship between immunohistochemical (IHC) subtypes of hepatocellular carcinoma vs. gene signatures, vs. tumor microenvironments (TMEs), and vs. clinicopathological features. It has been shown that the amount of collagen and elastin within hepatocellular carcinoma is large in B/S type, and less in −/−, or W/B types [48]. B/S, biliary/stem cell; W/B, Wint/β-catenin; −/−, both B/S and W/B negative; LGR5, Leucine-rich repeat-containing G-protein coupled receptor 5; OATP1B3, organic anion transporting polypeptide 1 B 3; EOB, ethoxybenzyl diethlenetriamine pentaacetic acid; poly7, polysomy of chromosome 7; CTNNB1, βcatenin gene; KRT19, keratin 19; EpCAM, epithelial cell adhesion molecule; TGF-β, transforming growth factor-β; Sci, scirrhous; SH, steatohepatitis; AFP, alpha-fetoprotein; VP, portal venous invasion; +++, severe; +, mild. Reproduced from reference [53] with permission.

In a sub-analysis of our previous study [24], we investigated which pathological factors are significantly related to ECV_HCC_, and multivariable analysis revealed that positive inflammatory cell infiltration, negative fibrous capsule, and positive scirrhous component are the independently significant factors as determinants of ECV_HCC_ (unpublished data). Independently, Maehara et al. [48] reported that higher amounts of intratumoral collagen and elastin were significantly associated with certain macroscopic types (confluent multinodular type rather than simple nodular type), positive intratumoral fat, and immunohistochemical subtypes (biliary-stem cell type, rather than Wint-βcatenin or both negative types) [51,52,53,54,55], in addition to positive inflammatory cell infiltration, negative fibrous capsule, and positive scirrhous component—factors completely overlapping with those related to ECV_HCC_ in our sub-analysis. Although our pathologists do not routinely perform immunohistochemical staining for HCC, and therefore we do not have direct evidence that ECV_HCC_ is related to immunohistochemical subtypes, the similarity in features, as seen on routine hematoxylin and eosin staining suggests that ECV_HCC_ may indeed correlate with immunohistochemical subtypes of HCC. Because immunohistochemical subtypes have been reported to be closely related to gene signatures, immunological tumor microenvironment, and clinicopathological features of HCC (Figure 7) [51,52,53,54,55], these observations may suggest the possibility that ECV_HCC_ can be used in the future as an indicator of biological aggressiveness of HCC, or as a predictor of response to systemic therapy such as immune checkpoint inhibitor or anti-VEGF agents.

Recently, Wang et al. [56] reported that the ECV of HCC may be used to predict Ki-67 positivity of HCC. Ki-67 expression usually indicates high proliferative status of malignant tumors, and as a result, biological aggressiveness of the neoplasm. Their observation that higher ECV may suggest a higher likelihood of Ki-67 expression is quite in accordance with our above-mentioned speculation. Li et al. [57] also reported machine learning models including the ECV of HCC can predict its histological grades (high vs. low grade). Thus, evidence has been accumulated suggesting that ECV, an important determinant of washout status at the EqP, is closely related to histological grades or the biological aggressiveness of HCC. Regarding Ki-67, however, Deng et al. [58] has reported the opposite result; namely, Ki-67 expression was inversely related to the ECV of HCC. Further investigation would be needed to solve this contradiction. Further, Fu et al. [59] reported that higher ECV of HCC is related to longer progression-free survival and overall survival in patients receiving immune checkpoint inhibitors. This is in accordance with previous observation, because higher ECV is associated with the biliary/stem cell immune histochemical subclass [51,52,53,54,55], which is immune hot compared to the other two subclasses (Figure 7) [51,52,53,54,55]. The correlation between the ECV of HCC and patient survival after chemoembolization has also been reported [60], but with equivocal results.

With respect to scanning protocol parameters, the delay times used for the EqP varied substantially among the studies cited here [24,29,30,31,32,33,34,35,36,37,38,39,40,41,42,43,44,45,46,47,56,57,58,59,60], ranging typically from about 120s to 300s. Indeed, in the above-mentioned contradictory reports regarding the relationship between ECV of HCC and Ki-67 expression reported by Wang et al. [56] and Deng et al. [58], the delay times for the EqP are significantly different: 200 s in the former [56] and 127 s in the latter [58]. A too short delay time for the EqP might have contributed to contrasting results, meaning that there was insufficient time for contrast equilibration between the IVS and EES. There is, as yet, no universally accepted definition for the optimal EqP delay time of the liver, but one author [34] has suggested that an EqP delay time of 180 s may be too short when considering the conceptual requirement of the “equilibrium” phase, where the iodine concentration should be equal in IVS and EES; under such circumstances, vessels within and around the liver, namely, the artery, portal vein, and hepatic vein, should exhibit the same or comparable density, which very rarely occurs when using a 180 s delay time. This issue should be thoroughly clarified in future studies.

## 3. Discussion and Summary

Washout at the PVP and that at the EqP are two distinct imaging phenomena, representing dynamic vs. more static intratumoral pathophysiological conditions, respectively. In practical terms, for radiologists working in daily clinical practice, a positive PVP washout may be considered as a sign of relatively higher grades of malignancy, the presence of MVI, poorer post-surgical survival, or inferior response to systemic therapy [19,20,21,22,23,26,27,28]. In contrast, negative EqP washout, which is an indirect marker of elevated ECV_HCC_, may suggest a biliary/stem cell subtype which is biologically aggressive, and relatively immune hot status, as opposed to the Wnt/β-catenin subtype or both negative subtypes, which are less aggressive and immune cold [48,56,57,59]. In other words, when washout is clearly seen on the PVP, or when you do not see clear washout on the EqP in daily clinical practice, the tumor is more likely to be at higher grade (poorly differentiated HCC), rather than at lower grade (well differentiated HCC). Thus, despite their different pathophysiological bases, both types of washout are closely related to clinico-pathological features, immuno-histochemical subtypes, and potentially even genetic signatures of HCC. Because of these associations, they hold considerable promise as noninvasive imaging biomarkers to predict tumor aggressiveness, postoperative survival, and response to systemic therapies.

It remains essential to assess how scanning parameters might influence these observations, for example, delay times used to acquire PVP/EqP [19,20,21,22,23,24,25,26,27,28,29,30,31,32,33,34,35,36,37,38,39,40,41,42,43,44,45,46,47,56,57,58,59,60,61], as well as recent low-kV/dual energy protocols [35,36,37,38,39,45,62]. Particularly, as discussed in Section 2 and Section 3, the difference in the delay times for the PVP/EqP may materially affect the performance of “washout” findings when used as predictors for patient outcomes. Therefore, technical aspects of imaging acquisition should be scrutinized carefully, and ideally be standardized to allow for generalization of the results across centers or countries.

In addition to washout, other imaging findings are emerging as potentially useful biomarkers to predict patient outcomes. For instance, the signal intensity features of HBP of EOB-MR have been reported to be closely related to the Wint/β-catenin positive subtype of HCC, which is associated with less biological aggressiveness, better prognosis, and often weaker response to immunotherapy [63,64,65,66]. Furthermore, the presence of intratumoral fat, as measured by chemical-shift imaging (CSI) of MRI, has been linked with steatohepatitic condition, which is the immuno-exhausted status of HCC [67].

Going forward, with an adequate combination or integration of PVP and EqP washout information, along with HBP and CSI information, it may be possible to develop predictive models, such as a nomogram or a weighted scoring system, that enables precise prediction of tumor aggressiveness or post-treatment outcomes. These models might be based on routine CT or MRI examination findings, making them broadly applicable in clinical practice.

## 4. Conclusions

PVP washout likely reflects intratumoral perfusion dynamics (i.e., flow rate), whereas EqP washout appears to be more closely tied to the amount of stroma and extracellular content within the tumor. Nevertheless, both are strongly associated with biological aggressiveness, and the tumor microenvironment of HCC, and thereby linked to response to systemic therapies and post-treatment outcome.

When information on both washout types is adequately integrated with other clinico-radiological features, such as hepatobiliary phase imaging and CSI information, even more precise prediction of survival and response to systemic therapies would become feasible in the future.
